# Genetic Variation in Damaged Populations of *Pistacia atlantica* Desf.

**DOI:** 10.3390/plants9111541

**Published:** 2020-11-11

**Authors:** Amina Labdelli, Roberto De La Herrán, Rami Arafeh, Francesca Resentini, Livio Trainotti, Youcef Halis, Ahmed Adda, Othmane Merah

**Affiliations:** 1 Scientific and Technical Research Centre for Arid Areas (CRSTRA), BP 1682 RP, Biskra 07000, Algeria; aminalabdelli@yahoo.fr (A.L.); youcefhal@gmail.com (Y.H.); 2 Laboratory of Agro-Biotechnology and Nutrition in Semi-Arid Areas, Ibn Khaldon University, Tiaret 14000, Algeria; adda2ahmed@yahoo.fr; 3 Departamento de Genética, Facultad de Ciencias, Universidad de Granada, Avda, Fuentenueva s/n, 18071 Granada, Spain; 4 Palestine-Korea Biotechnology Center, Palestine Polytechnic University, P.O. Box (198), Hebron, Palestine; arafeh@ppu.edu; 5 Department of Biosciences, Università degli Studi di Milano, Via Giovanni Celoria 26, 20133 Milano, Italy; 6 Department of Biology, University of Padova, Viale Giuseppe Colombo 3, 35121 Padova, Italy; livio.trainotti@unipd.it; 7 Laboratoire de Chimie Agro-Industrielle (LCA), Université de Toulouse, INRA, INPT, 31030 Toulouse, France; 8 Département Génie Biologique, Université Paul Sabatier, IUT A, 32000 Auch, France

**Keywords:** Atlas pistachio, population analyses, endangered species, genetic conservation, ISSR variation

## Abstract

The Atlas Pistachio tree, *Pistacia atlantica* Desf., has great importance in the ecological landscape of North Africa, due to its adaptive plasticity, as well as its use as a rootstock in the cultivation of the economically important species, *Pistacia vera* L. The conservation and valuation of this species require sampling and an assessment of its genetic variability. For the first time in North Africa, the inter-simple sequence repeats (ISSR) molecular marker has been used in genetic-diversity assessment and in the population relationships of *P. atlantica subsp. atlantica*. The ISSR markers tested showed 74.1% polymorphism, while molecular variance (AMOVA) analysis revealed a high percentage of the total genetic diversity of 55.7% among the four populations studied. Cluster analysis with neighbor-joining (NJ) and principal coordinate analysis (PCO) divided the study sites into four distinct groups according to their geographical locations (Tiaret, Batna, Djelfa, and Bechar). Isolation by distance or Mantel test gave a positive correlation of *r* = 0.86 between geographical and genetic distances. The results in this study indicate an absence of gene flow, implying that conservation efforts should be taken separately for each population.

## 1. Introduction

The genus *Pistacia,* Anacardiaceae family consists of at least 11 dioecious species [1], four of these being found in North Africa, including Algeria (*Pistacia vera* L., *P. terebinthus* L., *P. lentiscus* L., and *P. atlantica* Desf.). Pistachio (*P. vera* L.) is known for its economic value in agriculture as a nut crop [2,3,4]. The other three species possess ecological importance in addition to their use as medicinal plants and sources of feed for animals and birds [4]. The Atlas pistachio (*P. atlantica* Desf.) is used for its leaves and fruits that have high nutritional value, and the wood is also used in handicrafts, in carpentry, and for firewood [2,3,4,5]. *Pistacia atlantica* Desf. is an Irano-Turanian tree distributed from south-west Asia to north-west Africa [1]. There are four subspecies in *P. atlantica* have been recognized, subsp. *atlantica*, *kurdica*, *mutica*, and *cabulica*. The subsp. *atlantica* in North Africa is found in a wide distribution range from sub-humid environments to extremely arid Saharan sites [6]. Being xerophyte makes *P. atlantica* Desf. a prime choice for establishing pistachio cultivation in Algeria because it is used as a rootstock for the common pistachio crop [1]. This species plays important ecological roles because it combats soil erosion and prevents landslides when used for the reforestation of arid and steep slopes. Unfortunately, in many regions, *P. atlantica* is facing genetic erosion after being subjected to overgrazing, bush fires, deforestation, desertification, pollution, and other adverse factors. Consequently, it is listed among the endangered species in many regions [7,8]. The genetic relationships based on morphological or molecular markers have been performed on the genus Pistacia to determine their taxonomy [1,6,7,8,9,10,11,12]. Among the species of this genus, the Atlas pistachio tree is characterized by a remarkable morphological variability that allows it to adapt to different ecological conditions [6,7,8,9]. Based on this fact, it is urgent to proceed with an assessment of the genetic variability of this species to adopt the appropriate strategies for its preservation. It is becoming of greater importance to explore genetic diversity and population relationships of endangered plant species, especially those with slow growth and high morphological and biochemical variation [3,7,9], as in the case of Atlas pistachio.

Despite the importance of this species, very few genetic studies have been carried out on its populations or in relation to other *Pistacia* species [13,14,15]. Several molecular markers have been used to determine genetic diversity and relations among *Pistacia* species and cultivars. The randomly amplified polymorphic DNA (RAPD) was among the most commonly used methods in pistachio to study genetic diversity [13,16,17,18,19] for being quick and easy to develop. However, it lacks reproducibility—so today, the method has fallen into disuse. Ibrahim Basha et al. [20] detected high diversity in *P. vera* L. by amplified fragment-length polymorphisms (AFLP) markers in Syria—a country considered one of the centers of origin for this species. Other markers used in different studies in *Pistacia* ssp. [19,21,22,23] include the simple sequence repeats (SSR), which are specific and highly polymorphic markers [24], but require prior knowledge of the genomic sequence to design specific primers, and therefore, its development is limited to more economically important species, and in many cases, cross-amplification is ineffective. Furthermore, other methods have been used, such as the sequence-related amplified polymorphism (SRAP), the selectively amplified microsatellite polymorphic loci (SAMPL), and the inter-simple sequence repeats (ISSR) as well [15,25,26,27,28,29,30].

The molecular marker, called the inter-simple sequence repeat (ISSR), which provides high repeatability, has been used in systematic comparisons below the species level and varietal identification [20,27]. The technique does not require prior information about the genome sequence and leads to multi-locus and highly polymorphic patterns [25,26,27]. Also, it involves longer primers (16–18 nucleotides) encoding microsatellite elements that amplify DNA segment intra-microsatellite repeats [31]. Here, such a study is conducted for the first time on *P. atlantica* Desf. Algerian populations, based on the ISSR marker.

The aim of the present study was to examine the intraspecific genetic diversity of four populations of *P. atlantica* Desf. growing in different zones of Algeria. This is intended to provide insight into the relationships between the study populations and to provide data for the proper conservation of this tree, as well as for future selection programs of rootstock variability for pistachio cultivation.

## 2. Results

Two primer pairs (ISSR1 and ISSR 2) did not produce any polymorphic fragments with different annealing temperatures in the polymerase chain reaction (PCR) for any of the different *P. atlantica* Desf. samples. For the other two primer pairs, a total of 112 DNA fragments were amplified, 72 of which (64.3%) were polymorphic. The number of polymorphic fragments varied between the populations and the ISSR primers, showing a range from 4 (ISSR4 in Bechar) to 14 (ISSR3 in Tiaret). The percentage of polymorphic loci was 91% on average, and the highest degree of polymorphism was observed among wild populations of Tiaret (76.9%), whereas the lowest was found in the Djelfa population (50%; Table 1).

In the four natural populations of *P. atlantica* Desf. the number of effective alleles proved similar, ranging from 1.440 (Djelfa) to 1.64 (Tiaret). Expected heterozygosis and the genetic diversity parameter by Shannon’s information index (SI) values ranged from 0.233 (Djelfa) to 0.344 (Tiaret) and 3.6 (Bechar) to 5.34 (Djelfa), respectively (Table 2).

The analysis of the molecular variance (AMOVA) based on ISSR data showed high percentages of the total genetic diversity of *P. atlantica* Desf. populations distributed at this small spatial scale—with 55.7% among populations and 44.3% within populations. The pairwise Phi_ST_ value (coefficient: standard Jaccard distance transformation: d = 1 − s) was 0.56, indicating high differentiation among populations. Significant correlation between different populations was found in this study (*p* ≤ 0.001; pairwise Phi_ST_ values, standard Jaccard coefficient, distance transformation d = 1 − s) (B-group, D-group, T-group: 0.45 ***, 0.57 ***, 0.59 ***). An analysis using Structure software, performing runs from *K* = 2 up to *K* = 6, showed the genetic structure of the populations analyzed.

**Figure 1:**
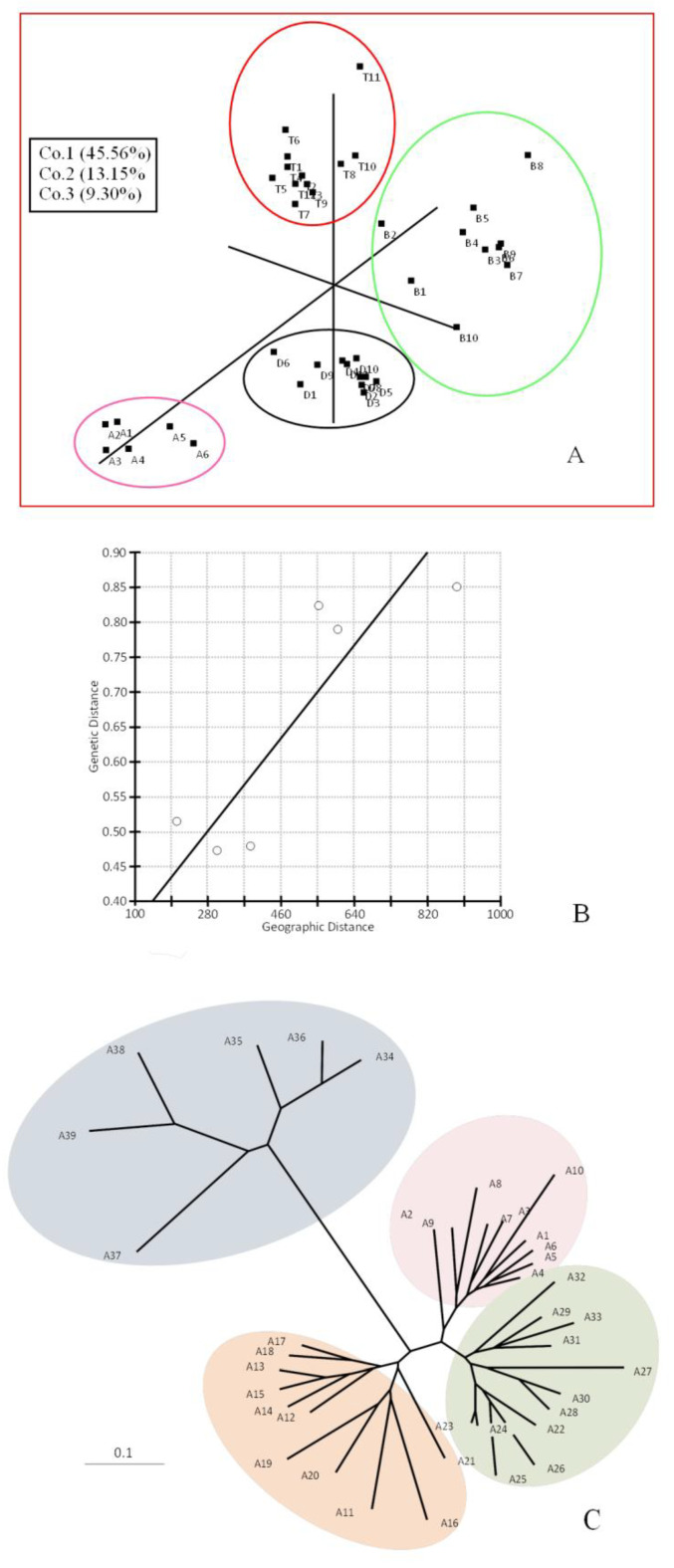
PCO Analysis, Correlation between genetic and geographic distances, and Midpoint rooted neighbor-joining tree. (**A**) Three-dimensional PCO analysis of ISSR data; (**B**) Correlation between genetic and geographic distances plotted with Z = 2119.05, *r* = 0.867 as calculated by the Mantel test; (**C**) Midpoint rooted neighbor-joining tree of samples from four populations of *P. atlantica* Desf. generated with ISSR data. Individuals from A1–A10 belong to the Batna population, A11–A21 to the Djelfa population, A22–A33 to the Tiaret population, and A34–A39 to the Bechar population.

**Figure 2:**
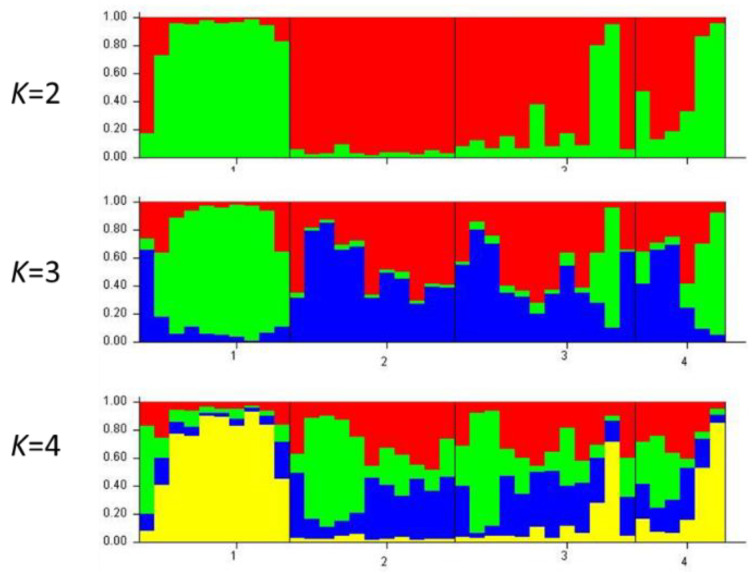
Graphical representation of the estimated membership coefficients for each individual obtained from the Bayesian clustering analysis of genetic structure for *K* = 2–4 computed from ISSR. Each individual is represented by a bar broken into *K* colored segments. The populations of Batna, Djelfa, Tiaret, and Bechar are indicated with the numbers 1 to 4, respectively.

The AMOVA approach also generates F_ST_ values (one of the F-statistics, such as Weir and Cockerham’s 98 measures [32], this parameter is calculated as the diversity among populations/total diversity. Values close to zero indicate little differentiation among populations (signifying mostly the genetic diversity within populations), while values close to one indicate high differentiation among populations. The principal coordinate analysis (PCO) clearly showed the four main groups from distinct geographical locations, Batna (B), Djelfa (D), Bechar (A), and Tiaret (T) (Figure 1A). Positions of the individual samples showed an overall homogeneity within the sampled site. On the other hand, isolation by distance or the Mantel test gave a positive correlation (*r* = 0.86, *p* = 0.131; Figure 1B). This positive r-value would indicate a certain degree of genetic isolation within a very narrow scale of the entire distribution range and also an indicator for genetic isolation between the four populations studied with an absence of gene flow between them, although they represent a narrow area at the eastern portion of the *P. atlantica* Desf. distribution range (Figure 1B). The ISSR data, used to find genetic relatedness among the genotypes, clustered the samples into four groups. Our results also indicated that *P. atlantica* Desf. subsp. *atlantica* clustering was related to the geographical locations and climatic conditions of its growing regions (Figure 1C). As a complementary study, an analysis using Structure software, performing runs from *K* = 2 up to *K* = 6, showed the genetic structure of the populations analyzed. The estimated membership coefficients for each individual (Figure 2) is represented according to differentiation among populations, where different genetic structures for the four populations in different degrees can be observed.

## 3. Discussion

Due to the lack of genomic information of this species, these markers are highly useful, since they require no prior knowledge of the sequences, and therefore, the primers need not be site-specific because they are directed to a genomic region containing a complementary microsatellite motif [33,34]. By means of the ISSR analysis, which has proved to be simple and efficient with high reproducibility, we found pronounced genetic variation in Atlas pistachio populations among the samples studied, i.e., enough to characterize the different Algerian populations analyzed. Thus, the ISSR technique proved adequate for discriminating different *P. atlantica* Desf. populations. Both of the ISSR primers used here generated polymorphisms and proved sufficient to discriminate all four populations. The principal coordinate analysis (PCO) and neighbor-joining (NJ) analysis yielded congruent patterns in the determination of genetic differences between the populations that are grown in different regions in Algeria. This finding suggests the influence of climatic conditions and geographic distances on the diversity of different sites of *P. atlantica* Desf.

**Figure 3:**
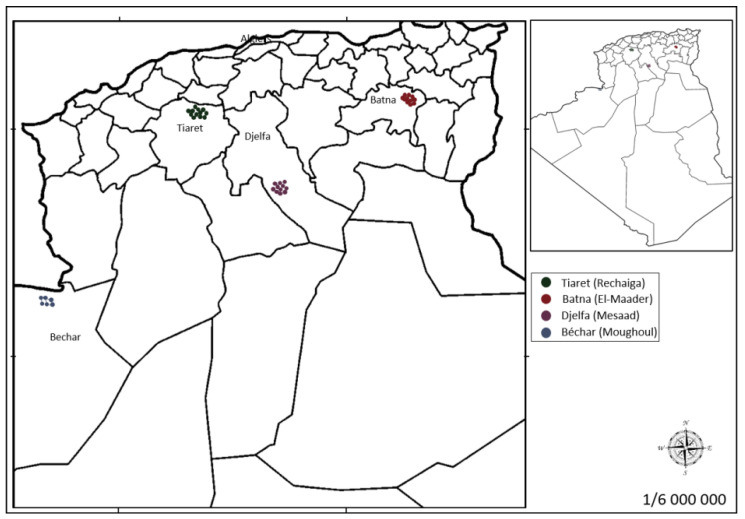
Geographical locations of the four sampled populations of *P. atlantica* Desf. collected in Algeria. The distance between them are: Batna (B)-Tiaret (T) ~387 Km; Batna (B)-Djelfa (D) ~300 Km; Batna (B)-Bechar (A) ~899 Km; Tiaret (T)-Djelfa (D) ~198 Km; Tiaret (T)-Bechar (A) ~563 Km; and Djelfa (D)-Bechar (A) ~604 Km.

Moreover, El Zerey-Belaskri et al. [19], studying populations of this species in north-western Algeria, demonstrated that a certain genetic similarity was established between them through evaluation by the SSR marker. According to these authors, this genetic linkage would occur through the dissemination of pollen grains and/or seeds between geographically close populations. This data supports the results obtained in our study, since the populations studied by El Zerey-Belaskri et al. [19], were distributed along a northwest-northeast transect of about 230 km, while the populations studied here are at much greater geographic distances, preventing the most cases a genetic flow between them (Figure 3).

The genetic relatedness among the genotypes was investigated by neighbor-joining (NJ) cluster analysis. This grouped the genotypes into four major groups with increasing aridity: low and medium aridity (T: Tiaret, B: Batna, D: Djelfa) and high aridity (A: Bechar) (Figure 1C). It bears noting that the most severe environmental conditions found in Bechar correlated with the highest genetic diversity within individuals of this population. For this population, the genetic diversity indexes also were similar to the other populations when they were compared (Table 2). These results corroborate that the ISSRs are good markers to detect genetic variation among closely related variants and in crop classification [29]. Mediterranean forests, in general, tend to be poor in endemic species [35]. In Algeria, the climate is transitional between maritime in the north and semi-arid to arid in the middle and the south [6]. Bechar is located in the south-west of Algeria, characterized by an arid climate with Saharan tendency and irregular precipitation. The interaction of Mediterranean and Saharan flora includes very high diversity, and therefore, deserves special conservation [8]. The high morphological and biochemical plasticity of *P. atlantica* Desf. in response to aridity may explain its wide ecological distribution in North Africa [6,7]. In this region, the ecological upheaval caused by deforestation and overgrazing has given rise to a serious dysfunction, which affects high-altitude forests in particular. Conservation of forests and forest species in the Mediterranean basin is a complex issue, given the wide range of ecological situations, diverse forest uses, and the pressure exerted by different cultural groups [35]. Based on the results of this study, conservation efforts should be focused on each population separately, due to the genetic isolation and absence of gene flow between the four populations. Additionally, the information on the genetic variability of these populations may be of interest at the time of selecting the specimens used as rootstocks in the cultivation of *P. vera* L.

## 4. Materials and Methods

### 4.1. Plant Materials

**Figure 4:**
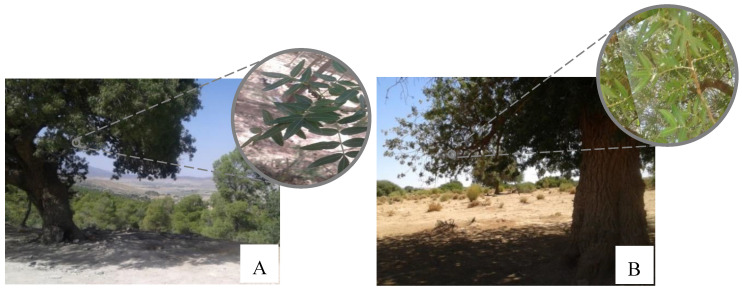
*Pistacia atlantica* Desf. *atlantica* tree and leaves in regions of Batna (**A**) and Bechar (**B**) (September 2015). Photos: Labdelli A.

Leaf samples of 39 *P. atlantica* Desf. trees were collected from four different locations in Algeria (Table 3; Figure 3 and Figure 4). A voucher specimen was deposited in the herbarium of the Scientific and Technical Research Centre for Arid Areas (CRSTRA), Biskra, Algeria (006CRSTRA0002). Details of the ecological factors for each collection site are reported in Labdelli et al. [3].

### 4.2. DNA Extraction

The leaf samples were dried by silica gel and stored at −80 °C until used. Genomic DNA was extracted from leaf tissue by the CTAB (hexadecyltrimethyl ammonium bromide) method with the modifications adopted by Kafkas [36] and Al-Sousli et al. [37]. Young leaf tissues (100 mg) was manually ground into a fine powder in liquid nitrogen with a plastic pestle in a 2.0-mL Eppendorf tube and mixed with 900 µL of CTAB extraction buffer (1 M TRIS-HCl pH 8, 5 M NaC1, 0.5 M EDTA, 2% CTAB, 2% polyvinylpyrrolidone PVP, 0.2% b-mercaptoethanol, and 0.1% NaHSO_3_).

Samples were incubated at 65 °C for 1 h, mixed with an equal volume of chloroform-isoamyl alcohol (24:1), and centrifuged for 5 min at 13,000 rpm. The aqueous phase was transferred to a new 2-mL Eppendorf tube, mixed with equal volume of ice-cold isopropanol to precipitate the DNA, and left at −80 °C for 1 h. The tubes were gently inverted several times. The nucleic acid pellet was recovered by centrifugation at 13,000 rpm for 5 min, washed with 1.0 mL of 10.0 mM ammonium acetate in 70% ethanol for a few min, dried for 15 min at room temperature, and resuspended in 100 µL modified TE buffer pH 8.0 (10 mM TRIS-HCI, 0.l mM EDTA) added with RNAase (5 µg/mL). The concentration of the DNA was estimated by comparing band intensity with a DNA of known concentrations on 0.8% agarose gel in 1× TAE buffer. The DNA was diluted to 20 ng/µL for ISSR reactions.

### 4.3. ISSR-PCR Amplification

The samples were genotyped for ISSR markers using GC- and CA-rich primers. Amplifications were performed in 25 µL reaction mixture containing 1.7 µL of genomic DNA (20 ng), 0.8 µL primer (Table 4), 4.0 µL of reaction buffer (5× Green GoTaq^®^ Reaction Buffer), 0.8 µL of MgCl_2_ (50 mM) (or DMSO dimethyl sulfoxide + buffer 10× PFU Reaction w/20 Mm MgSO_4_ Promega), 0.8 µL dNTPs, 0.2 µL of Taq DNA Polymerase (PFU DNA Polymerase 5 U/µL), 11.7 µL of high-performance liquid chromatography (HPLC) grade water. The thermocycler program included 1 cycle of initial DNA denaturation at 94 °C for 10 min, followed by 35 cycles of 45 s at 94 °C, 45 s annealing at 54 °C and 2 min extension at 72 °C, followed by a final extension for 10 min at 72 °C [37]. PCR reactions were run in agarose gel (3.0% *w*/*v*) in 1× TAE buffer and visualized by UV light.

### 4.4. Data Analysis

The ISSR amplicons (bands) were scored as present (1) or absent (0) according to their size, and then a binary data matrix was constructed. Only clear and reproducible bands were scored and used in the data analysis. Bands were assumed to be independent, and those of identical size were assumed to have identical sequences. The genetic diversity indices, including the number of alleles (Na), percentage of polymorphic loci (%P), number of different alleles (Nd), number of effective alleles (Ne), expected heterozygosity (He), and Shannon’s information index (SI), were assessed at each population levels using FAMD Software version 1.31 [38] and GenAlEx Software [39].

The similarity matrix was also used to perform a hierarchical analysis of molecular variance (AMOVA), according to Excoffier et al. [40] using fingerprinting analysis with missing data (FAMD) Software version 1.31 [38]. This analysis enables the partitioning of the total ISSR variation into within and among geographical region variation components (called Phi statistics).

The genetic structure of populations was simulated by a Bayesian clustering analysis with the software STRUCTURE, version 2.3 [41]. The genotypic data of populations was analyzed under a clustering model of admixture of individuals with correlated allele frequencies among populations. Analysis of ISSR data included a model of clusters from *K* = 2 to *K* = 4.

The genetic distance matrix was estimated based on Jaccard’s similarity coefficient using the multi-locus fingerprinting data sets containing missing data [FAMD software, version 1.31]. A cluster analysis was performed using the neighbor joining (NJ) method with arithmetic averages and viewed with Tree View software (Win32, version 1.6.6). PCO analysis was made with FAMD version 1.3 (Fingerprinting analysis with missing data) using the Jaccard’s similarity index [42].

To test for isolation by distance [40], pairwise log_10_ transformed values of N_e_^m^ among populations were linearly regressed against their geographical (=aerial map) distances (in km). The significance of the regression slopes was evaluated by Mantel’s test [43] with 10,000 random permutations using the Isolation by Distance Web Service version (3.23) under the link http://ibdws.sdsu.edu/~ibdws.

## 5. Conclusions

This work shows that Algeria still contains a rich genetic diversity for the Atlas Pistachio, which is likely to be valued. Despite the strict allogamy of this species and its highly dynamic gene flow, the geographical remoteness of the study areas indicates that this species still maintains a diversity of genetic entities. This inter-population diversity would certainly be remodeled by natural selection under the diversity of the environmental conditions of the different prospected areas. The results also indicate that the rate of intra-population genetic variability is negatively correlated with the increase in density of the populations studied.

## Figures and Tables

**Table 1:** The number of amplified and polymorphic bands observed at the different locations. ISSR, inter-simple sequence repeats.

Primer		Batna	Djelfa	Tiaret	Bechar
**ISSR3**	Total number of bands	19	14	19	13
	Number of polymorphic bands	13	7	14	9
	% polymorphic bands	**68.42**	**50**	**73.68**	**69.23**
**ISSR4**	Total number of bands	14	14	13	6
	Number of polymorphic bands	8	7	10	4
	% polymorphic bands	**57.14**	**50**	**76.92**	**66.67**

**Table 2:** Genetic diversity of the four populations of *P. atlantica* Desf. inferred by the two ISSR markers.

Populations	*Na*	*%P*	*Nd*	*Ne*	*He*	*SI*
**Batna**	83	63.64%	1.636 ± 0.085	1.504 ± 0.076	0.272 ± 0.039	5.26 ± 0.29
**Djelfa**	83	50%	1.500 ± 0.096	1.440 ± 0.086	0.233 ± 0.045	5.34 ± 0.36
**Tiaret**	83	75%	1.750 ± 0.078	1.640 ± 0.069	0.344 ± 0.036	5.32 ± 0.17
**Bechar**	33	68.42%	1.684 ± 0.110	1.565 ± 0.071	0.305 ± 0.051	3.6 ± 0.19

*Na*: Number of alleles; *%P*: Percentage of polymorphic loci; *Nd:* Number of different alleles; *Ne*: Number of effective alleles; *He*: Expected heterozygosity; and *SI*: Shannon’s information index.

**Table 3:** Geographical locations and numbers of samples collected from each *P. atlantica* Desf. population analyzed.

Assigned Code	Location	Number of Samples	Altitude (m)	Latitude (N)	Longitude
**B**	Batna Semi-arid	10	1027	35°37′10″	6°22′13″ E
**T**	Tiaret Semi-arid	12	808	35°22′33″	02°09′5″ W
**D**	Djelfa Arid	11	630	34°02′11″	03°40′22″ E
**A**	Bechar Hyperarid	06	979	32°04′6″	02°18′5″ W

**Table 4:** List of the ISSR primers used in this study, their sequences, and the annealing temperature applied to each. (W= A or T, Y= C or T, B= T, or C or G).

Primer Name	Sequence 5′–3′	Annealing Temperature (°C)
ISSR 1	(CG)_9_W	From 50 to 60
ISSR 2	(GC)_9_W	From 50 to 60
ISSR 3	(AG)_9_B	54
ISSR 4	(GA)_9_Y	54
